# Perceptions of health warnings on cigarette sticks among the adult population in Al-Madinah, Saudi Arabia: A cross-sectional survey

**DOI:** 10.18332/tid/182912

**Published:** 2024-02-21

**Authors:** Ahmed F. Al-Ahmadi, Mohammed A. Almatrafi, Ahmed K. Ali, Osama H. Alsaedi, Abdulmohsen H. Al-Zalabani

**Affiliations:** 1 General Directorate of Health Affairs of Medina, Ministry of Health, Al Madinah, Saudi Arabia; 2 Model of Care, Madinah Health Cluster, Al-Madinah, Saudi Arabia; 3 Quit Smoking Clinic, Public Health Department, King Salman Medical City, Al-Madinah, Saudi Arabia; 4 Preventive Medicine Program for Postgraduate Studies, Ministry of Health, Al-Madinah, Saudi Arabia; 5 Department of Family and Community Medicine, College of Medicine, Taibah University, Al Madinah, Saudi Arabia

**Keywords:** Saudi Arabia, public health, tobacco control, dissuasive cigarettes

## Abstract

**INTRODUCTION:**

Health warnings on cigarette sticks are emerging as a tool to control tobacco consumption; hence, understanding how they are perceived is valuable in determining their effectiveness. Our study aimed to evaluate the perception of health-related messages and warnings on individual cigarette sticks. It also aimed to evaluate the perceptions of the effectiveness of cigarette packaging warnings and the acceptance level for the inclusion of health warnings on cigarette sticks.

**METHODS:**

This cross-sectional survey was conducted on 285 individuals in Al-Madinah, Saudi Arabia. The survey was distributed online using a non-probability convenience sampling technique. The chi-squared test and logistic regression analysis were used to determine the association of sociodemographic characteristics and smoking-related variables with participants’ perceptions of health warnings on cigarette sticks and packaging. The responses were also assessed qualitatively using conceptual content analysis.

**RESULTS:**

In all, 18.6% of participants perceived that the package warnings were either ‘quite effective’ or ‘very effective’ in prompting smokers to quit. For health warnings on cigarette sticks, 28.1% of participants perceived that the theme of statistics on mortality was either ‘quite effective’ or ‘very effective’ in prompting smokers to quit, compared to 35.0 % for the theme of social and financial consequences. Respondents who had secondary education and lower were almost two times more likely to support the inclusion of health warnings than those who had a university education and higher (OR=1.9; 95% CI: 1.02–3.7, p=0.042). Most of the comments were positive for the inclusion of health warnings on cigarette sticks.

**CONCLUSIONS:**

Most participants perceived that package warnings were ineffective, but warnings on cigarette sticks were effective methods of dissuasion of cigarette use. Smokers were almost twice as likely to perceive as effective supportive messages to quit than non-smokers. The majority of participants ‘agreed’ or ‘strongly agreed’ to the inclusion of health warnings on cigarette sticks.

## INTRODUCTION

Smoking remains the leading cause of preventable diseases and death around the world^1^. Although the majority of smokers regret smoking^2,3^, their cessation intentions are affected by many factors, especially public awareness of the negative health effects of smoking^4,5^ and the financial burden of smoking^1,4^. Messages illustrating the health impacts of smoking are mostly delivered in developed countries in the form of health warnings on cigarette packages and media campaigns^6,7^.

The minimum recommendations for such public health interventions are set out by the Framework Convention on Tobacco Control (FCTC) published by the World Health Organization^8^. Section 11 of the FCTC outlines the recommendations for tobacco product labeling and packaging, mentioning the use of textual and illustrated warnings, the use of plain packaging, and the elimination of deceptive brand elements^8^. Many published articles have concluded that interventions in the field of tobacco packaging have helped to fill knowledge gaps regarding the hazards of tobacco use and improved public perception of and sensitivity toward the consequences of tobacco use^7,8-14^. Moreover, there is an abundance of health-related messages that enhance the confidence of tobacco users in smoking cessation and support its advantages^13^.

Although these public health interventions have generally resulted in a significant reduction in tobacco consumption over time, they can become less effective because of the frequent exposure of smokers to these warning messages throughout the year^14-16^. Recent studies have mentioned the use of the single cigarette stick as a useful tool to deliver health warning messages regarding tobacco consumption, which can enhance the use of cigarette packaging warnings^17-22^.

To the best of our knowledge, no studies have reported on the use of health warning messages on cigarette sticks in Saudi Arabia or the Middle East region. Our study aims to evaluate the perception of health-related messages and warnings on individual cigarette sticks and to identify the most effective messages on cigarette sticks. Additionally, we aim to evaluate perceptions of the effectiveness of cigarette packaging warnings, and explore both the positive and negative aspects of controlling tobacco consumption. Finally, we aim to evaluate the acceptance level for the inclusion of health warnings on cigarette sticks.

## METHODS

### Study design

A cross-sectional survey was conducted in Al-Madinah, Saudi Arabia, in June 2023. Online Google surveys were distributed irrespective of smoking status through WhatsApp by using a non-probability convenience sampling technique. Data collectors sent invitations to the target population until the minimum required sample size was achieved. Invitations were sent according to the following inclusion criteria: 1) adults aged ≥18 years, 2) smokers and non-smokers, 3) can read Arabic, and 4) registered in Madinah Health Cluster and can access all primary healthcare centers in Al-Madinah. Individuals who refused to participate were excluded from the study. The minimum required sample size was calculated to be 237 using the OpenEpi calculator tools website with the following assumptions: the estimated disagreement of the inclusion of health warnings was 19%, with 5% confidence limits, and a 95% confidence level.

### Study instrument and data collection procedure

Both qualitative and quantitative data were collected using a self-administered online questionnaire. The first part of the questionnaire covered demographic data (age, gender, nationality, residence, marital status, education level, and current position) and questions related to smoking (smoking status, type of tobacco products used, intention to quit smoking, perception of smoking harms). In the second part, the perceived effectiveness of both cigarette packaging warnings and cigarette stick warnings was assessed on a 5-point Likert scale ranging from ‘Not at all effective’ to ‘Very effective’. To determine the acceptance level, all the participants were asked for their opinions on the implementation of health warnings on cigarette sticks on a 5-point Likert scale ranging from ‘Strongly disagree’ to ‘Strongly agree’^23,24^. The questionnaire was checked by experts in the field for face validity. It was also pilot-tested to check clarity and acceptability.

**Figure 1:**
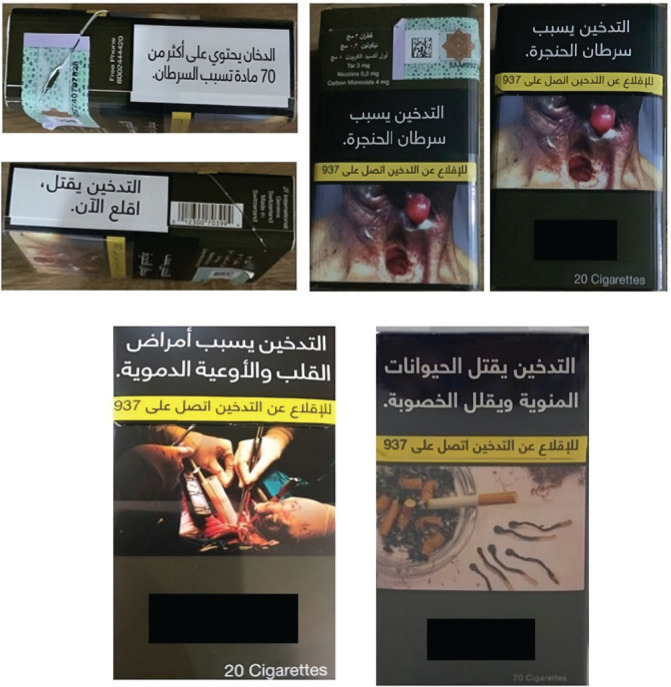
Warning messages are shown on the front, sides, and back of commonly circulated cigarette packages in Saudi Arabia: A cross-sectional survey, Al-Madinah, 2023 (N=285)

**Figure 2:**
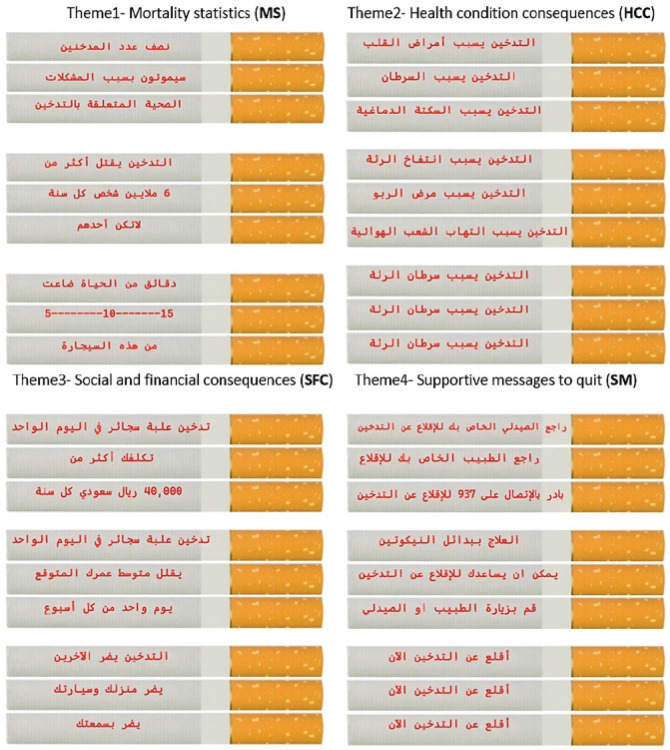
The health warnings included on cigarette sticks were grouped into four themes (three warnings are included in each theme): A cross-sectional survey, Al-Madinah, 2023 (N=285)

The data collector sent an invitation to eligible participants according to the inclusion criteria of the study. After the completion of demographic-related questions by all participants and smoking-related questions by smokers, photographs of commonly circulated cigarette packaging warnings in Saudi Arabia were displayed on the screen for each participant (Figure 1). The perceived effectiveness was then assessed using two questions on a 5-point Likert scale as mentioned above. The first question assessed the perceived effectiveness of the message in persuading smokers to quit, and the second question assessed its potential for preventing non-smokers from starting smoking. After that, 12 photographs of health warnings on cigarette sticks were displayed on the screen. Each cigarette contained three lines of health warnings, when rotated. The health warnings on the cigarette sticks were grouped into four themes: statistics on mortality, health consequences, social and financial consequences, and supportive messages to quit smoking. The health messages were adapted from Drovandi et al.^24^ and translated into Arabic (Figure 2). Further details of the health warnings on cigarette sticks have been explained in English in previous studies^23,24^. The perceived effectiveness of each health warning message was assessed by two questions on a 5-point Likert scale, similar to those regarding health warnings on cigarette packaging. Finally, participants were asked to rank their agreement or disagreement concerning the addition of health warnings to individual cigarettes on a scale of 1 to 5 (from ‘Strongly disagree’ to ‘Strongly agree’). Comment boxes were incorporated into the cigarette packaging warnings section and for each theme used in the health warnings on cigarette sticks to acquire qualitative information that explained participants’ justifications for their evaluations. The participants took around 5–10 minutes to complete the questionnaire. Data collection was conducted in June 2023.

Regarding open-text comments, two authors (AFA and OHA) independently assessed the responses using conceptual content analysis to verify emerging themes^24,25^. To identify areas of convergence and divergence between the two datasets and integrate these findings into the conclusions, quantitative and qualitative data were triangulated, and discrepancies in interpretations were settled through discussion. Quotes were translated from Arabic into English using the Reverso website^25^. Translation was reviewed independently by two investigators.

### Statistical analysis

A descriptive analysis of the demographic data was used to evaluate the characteristics of the study population. Data were entered via IBM Corp. Released 2017. IBM SPSS Statistics for Windows, Version 25.0. Armonk, NY: IBM Corp. The chi-squared test was used to determine the association of sociodemographic and smoking-related variables with participants’ perceptions of health warnings on cigarette sticks and packaging. Ordinal logistic regression analysis was checked for assumption and used to determine the perceived effectiveness of packaging warnings and cigarette stick warnings against smoking; the model was not adjusted, and only smoking status was used as an independent variable. Multivariable logistic was used, and adjusted odds ratios (AOR) and 95% confidence intervals were also calculated to assess participants’ perceptions of the health warnings and their acceptance of the inclusion of health warnings on cigarette sticks in relation to the collected variables. Confounders were adjusted for gender, employment status, age, marital status, education level, and smoking status, and selected based on plausibility and previous literature findings. All tests were two-tailed, and a p<0.05 was considered statistically significant.

Ethical approval was obtained from the research ethics committee of the General Directorate of Health Affairs of Al-Madinah City (Approval number: 23–024; Date: 19 March 2023). Informed consent was provided by the participants, and the data remained anonymous, with only the research team having access to them.

## RESULTS

Out of 540 participants who received the invitations, 285 completed the online survey (response rate: 53%). The mean age was 31 (SD=12.3) years, the median age was 25 years (IQR: 21–40), and the age range was 18–68 years. The majority of the participants were Saudi (98.2%), and living in Al-Madinah (92.3%). Nearly two-thirds (65.6%) of smokers perceived that smoking is very harmful to their health (Table 1).

**Table 1:** Sociodemographic and smoking-related characteristics of the participants: A cross-sectional survey, Al-Madinah, 2023 (N=285)

*Characteristics*	*All n (%)*	*Smokers n (%)*	*Non-smokers n (%)*
**Total**	285 (100)	68 (23.9)	217 (76.1)
**Age** (years), mean ± SD	31 ± 12.3	29.0 ± 11.1	31.7 ± 12.6
**Age** (years)			
18–25	144 (50.5)	38 (26.4)	106 (73.6)
26–45	101 (35.4)	22 (21.8)	79 (78.2)
46–65	38 (13.3)	8 (21.1)	30 (78.9)
≤66	2 (0.7)	0 (0.0)	2 (100)
**Gender**			
Male	143 (50.2)	60 (42.0)	83 (58.0)
Female	142 (49.8)	8 (5.6)	134 (94.4)
**Marital status**			
Single	155 (54.4)	42 (27.1)	113 (72.9)
Married	121 (42.5)	23 (19.0)	98 (81.0)
Divorced	9 (3.2)	3 (33.3)	6 (66.7)
**Education level**			
Illiterate	1 (0.4)	0 (0.0)	1 (100)
Did not complete primary education	1 (0.4)	0 (0.0)	1 (100)
Primary	3 (1.1)	2 (66.7)	1 (33.3)
Intermediate	6 (2.1)	2 (33.3)	4 (66.7)
Secondary	92 (32.3)	26 (28.3)	66 (71.7)
University and higher	182 (63.9)	38 (20.9)	144 (79.1)
**Employment status**			
Employed	131 (46.0)	35 (26.7)	96 (73.3)
Unemployed	123 (43.2)	29 (23.6)	94 (76.4)
Retired	15 (5.3)	4 (26.7)	11 (73.3)
Housewife	16 (5.6)	0 (0.0)	16 (100)
**Income** (SAR)			
<4000	144 (50.5)	30 (20.8)	114 (79.2)
4000–8000	54 (18.9)	20 (37.0)	34 (63.0)
>8000	87 (30.5)	18 (20.7)	69 (79.3)
**Perceptions of harm from smoking**			
Not at all harmful	1 (0.4)	0 (0.0)	1 (100)
Minimally harmful	4 (1.4)	3 (75.0)	1 (25.0)
Some harm expected	25 (8.8)	16 (64.0)	9 (36.0)
Quite harmful	68 (23.9)	28 (41.2)	40 (58.8)
Very harmful	187 (65.6)	21 (11.2)	166 (88.8)

SAR: 1000 Saudi Riyals about US$270.

### Current packaging warnings

More than half of the participants (52.3%) perceived that the warnings used on the packaging were either ‘ineffective’ or ‘minimally effective’, whereas 18.6% perceived that the warnings were either ‘quite effective’ or ‘very effective’ in prompting smokers to quit (Table 2).

**Table 2:** Ratings of the effectiveness of the current packaging warnings and novel cigarette stick warnings: A cross-sectional survey, Al-Madinah, 2023 (N=285)

*Warning methods*	*Ineffective n (%)*	*Minimally effective n (%)*	*Moderately effective n (%)*	*Quite effective n (%)*	*Very effective n (%)*	Effectiveness score* Mean (SD)
**All**						
**Packaging warnings**						
Preventing non-smokers from smoking	50 (17.5)	68 (23.9)	95 (33.3)	33 (11.6)	39 (13.7)	2.8 (1.2)
Prompting smokers to quit	71 (24.9)	78 (27.4)	83 (29.1)	22 (7.7)	31 (10.9)	2.5 (1.2)
**Statistics on mortality**						
Preventing non-smokers from smoking	20 (7.0)	51 (17.9)	95 (33.3)	64 (22.5)	55 (19.3)	3.2 (1.1)
Prompting smokers to quit	31 (10.9)	71 (24.9)	103 (36.1)	41 (14.4)	39 (13.7)	2.9 (1.1)
**Health consequences**						
Preventing non-smokers from smoking	18 (6.3)	58 (20.4)	84 (29.5)	73 (25.6)	52 (18.2)	3.2 (1.1)
Prompting smokers to quit	30 (10.5)	71 (24.9)	88 (30.9)	54 (18.9)	42 (14.7)	3.0 (1.2)
**Social and financial consequences**						
Preventing non-smokers from smoking	26 (9.1)	53 (18.6)	78 (27.4)	61 (21.4)	67 (23.5)	3.3 (1.2)
Prompting smokers to quit	37 (13.0)	74 (26.0)	74 (26.0)	54 (18.9)	46 (16.1)	2.9 (1.2)
**Supportive messages to quit**						
Preventing non-smokers from smoking	42 (14.7)	58 (20.4)	79 (27.7)	58 (20.4)	48 (16.8)	3.0 (1.2)
Prompting smokers to quit	42 (14.7)	63 (22.1)	84 (29.5)	46 (16.1)	50 (17.5)	2.9 (1.2)
**Smokers**						
**Packaging warnings**						
Preventing non-smokers from smoking	20 (29.4)	9 (13.2)	22 (32.4)	10 (14.7)	7 (10.3)	2.6 (1.3)
Prompting smokers to quit	19 (27.9)	17 (25.0)	17 (25.0)	7 (10.3)	8 (11.8)	2.5 (1.3)
**Statistics on mortality**						
Preventing non-smokers from smoking	10 (14.7)	16 (23.5)	19 (27.9)	10 (14.7)	13 (19.1)	3.0 (1.3)
Prompting smokers to quit	11 (16.2)	19 (27.9)	25 (36.8)	4 (5.9)	9 (13.2)	2.7 (1.2)
**Health consequences**						
Preventing non-smokers from smoking	9 (13.2)	17 (25.0)	17 (25.0)	15 (22.1)	10 (14.7)	3.0 (1.2)
Prompting smokers to quit	11 (16.2)	15 (22.1)	15 (22.1)	18 (26.5)	9 (13.2)	2.9 (1.2)
**Social and financial consequences**						
Preventing non-smokers from smoking	9 (13.2)	15 (22.1)	15 (22.1)	7 (10.3)	22 (32.4)	3.2 (1.4)
Prompting smokers to quit	14 (20.6)	12 (17.6)	16 (23.5)	7 (10.3)	19 (27.9)	3.0 (1.4)
**Supportive messages to quit**						
Preventing non-smokers from smoking	11 (16.2)	12 (17.6)	17 (25.0)	13 (19.1)	15 (22.1)	3.1 (1.3)
Prompting smokers to quit	13 (19.1)	11 (16.2)	18 (26.5)	5 (7.4)	21 (30.9)	3.1 (1.4)
**Non-smokers**						
**Packaging warnings**						
Preventing non-smokers from smoking	30 (13.8)	59 (27.2)	73 (33.6)	23 (10.6)	32 (14.7)	2.8 (1.2)
Prompting smokers to quit	52 (24.0)	61 (28.1)	66 (30.4)	15 (6.9)	23 (10.6)	2.5 (1.2)
**Statistics on mortality**						
Preventing non-smokers from smoking	10 (4.6)	35 (16.1)	76 (35.0)	54 (24.9)	42 (19.4)	3.3 (1.1)
Prompting smokers to quit	20 (9.2)	52 (24.0)	78 (35.9)	37 (17.1)	30 (13.8)	3.0 (1.1)
**Health consequences**						
Preventing non-smokers from smoking	9 (4.1)	41 (18.9)	67 (30.9)	58 (26.7)	42 (19.4)	3.3 (1.2)
Prompting smokers to quit	19 (8.8)	56 (25.8)	73 (33.6)	36 (16.6)	33 (15.2)	3.0 (1.1)
**Social and financial consequences**						
Preventing non-smokers from smoking	17 (7.8)	38 (17.5)	63 (29.0)	54 (24.9)	45 (20.7)	3.3 (1.2)
Prompting smokers to quit	23 (10.6)	62 (28.6)	58 (26.7)	47 (21.7)	27 (12.4)	2.9 (1.1)
**Supportive messages to quit**						
Preventing non-smokers from smoking	31 (14.3)	46 (21.2)	62 (28.6)	45 (20.7)	33 (15.2)	3.0 (1.2)
Prompting smokers to quit	29 (13.4)	52 (24.0)	66 (30.4)	41 (18.9)	29 (13.4)	2.9 (1.2)

* Effectiveness score using a 5-point Likert scale ranging from 1=‘Ineffective’ to 5=‘Very effective’.

### Novel warnings on individual cigarette sticks

First, regarding the theme of the statistics on mortality, the respondents perceived that the theme was ‘quite effective’ (22.5%) and ‘very effective’ (19.3%) in preventing non-smokers from smoking, higher than ‘quite effective’ (14.4%) and ‘very effective’ (13.7%) in prompting smokers to quit. Similarly, for the remaining themes (health consequences, social and financial consequences), except for supportive messages, themes were more frequently rated ‘effective’ in prompting smokers to quit than in preventing non-smokers from smoking (Table 2).

In bivariate analysis, respondents who were non-Saudi were more likely to perceive that the packaging warnings were effective compared to those who were Saudi (60% vs 15%, p=0.029), which could be attributed to the smaller sample size of non-Saudis. Those who had a secondary education perceived the themes to be more effective compared to those who had a university education and higher (22.3% vs 12.1%, p=0.023). Additionally, those who lived outside Al-Madinah compared to those who lived in Al-Madinah (45.5% vs 25.9%, p=0.048), and non-smokers compared to smokers (30.4% vs 17.6%, p=0.039), perceived the theme of the statistics of mortality to be more effective. Regarding the social and financial theme, those who had a secondary education level and lower (44.7% vs 28%, p=0.004) perceived the theme to be more effective. Finally, those who were smokers (41.2% vs 27.6%, p=0.035), non-Saudi (80% vs 30%, p=0.033), and lived outside Al-Madinah (59.1% vs 28.5%, p=0.003), were significantly more in perceiving that the supportive messages to quit were effective.

Regarding the statistics on mortality theme, smokers were less likely to perceive it as effective in preventing non-smokers from smoking compared to non-smokers (OR=0.5; 95% CI: 0.3–0.8; p=0.013). Moreover, they were less likely to perceive that it was effective in prompting current smokers to quit compared to non-smokers (OR=0.5; 95% CI: 0.3–0.9, p=0.040). Similarly, regarding the health consequences theme, smokers were less likely to perceive the theme as effective in preventing non-smokers from smoking compared to non-smokers (OR=0.5; 95% CI: 0.3–0.9; p=0.034) (Table 3).

**Table 3:** Perceived effectiveness of packaging warnings and cigarette stick warnings against smoking: A crosssectional survey, Al-Madinah, 2023 (N=285)

*Warning methods (Ref.: non-smokers)*	*OR*	*95% CI*	*p*
**Packaging warnings**			
Preventing non-smokers from smoking	0.9	0.5–1.5	0.849
Prompting smokers to quit	1.0	0.6–1.7	0.837
**Statistics on mortality**			
Preventing non-smokers from smoking	0.5	0.3–0.8	0.013*
Prompting smokers to quit	0.5	0.3–0.9	0.040*
**Health consequences**			
Preventing non-smokers from smoking	0.5	0.3–0.9	0.034*
Prompting smokers to quit	1.0	0.6–1.8	0.711
**Social and financial consequences**			
Preventing non-smokers from smoking	0.7	0.4–1.2	0.291
Prompting smokers to quit	1.1	0.6–1.8	0.673
**Supportive messages to quit**			
Preventing non-smokers from smoking	1.1	0.7–1.9	0.551
Prompting smokers to quit	1.1	0.7–1.9	0.491

* Statistically significant at p<0.05.

In the multivariable logistic regression, respondents who had secondary education were around two times more likely to perceive as effective the packaging warnings, health consequences, social and financial consequences, and supportive messages to quit (Table 4). In addition, unmarried women were more than two times more likely to perceive as effective the health consequences’ theme compared to married women (AOR=2.4; 95% CI: 1.0–5.5; p=0.038). Smokers had 60% lower odds of perceiving the statistics on mortality as effective compared to non-smokers (AOR=0.4; 95% CI: 0.2–0.9, p=0.046), whereas smokers were almost twice as likely to perceive the supportive messages to quit as effective compared to non-smokers (AOR=1.9; 95% CI: 1.0–3.7, p=0.047) (Table 4).

**Table 4:** Multivariable logistic regression analysis of the perceived effectiveness of packaging warnings and cigarette stick warnings: A cross-sectional survey, Al-Madinah, 2023 (N=285)

*Variables*	*Packaging warnings AOR (95% CI)*	*Statistics on mortality AOR (95% CI)*	*Health consequences AOR (95% CI)*	*Social and financial Consequences AOR (95% CI)*	*Supportive messages to quit AOR (95% CI)*
**Age** (years)					
18–25 ®	1	1	1	1	1
26–45	1.1 (0.3–3.6)	1.6 (0.5–5.0)	3.4 (1.3–9.1)*	1.2 (0.4–3.1)	1.8 (0.7–4.9)
≥46	1.1 (0.3–4.4)	0.9 (0.5–1.7)	**2.9 (0.9–8.8)**	1.2 (0.4–3.7)	1.4 (0.4–4.3)
**Gender**					
Male ®	1	1	1	1	1
Female	1.3 (0.6–2.7)	0.9 (0.5–1.7)	0.9 (0.5–1.6)	0.7 (0.4–1.3)	1.1 (0.6–2.0)
**Marital status**					
Married ®	1	1	1	1	1
Unmarried	1.0 (0.3–2.8)	1.0 (0.4–2.5)	2.4 (1.0–5.5)*	0.6 (0.2–1.5)	0.7 (0.3–1.8)
**Education level**					
University education and higher ®	1	1	1	1	1
Secondary education and lower	2.5 (1.1–5.2)*	1.5 (0.8–2.9)	2.0 (1.1–3.7)*	2.5 (1.3–4.4)*	2.1 (1.1–3.9)*
**Employment**					
Employed ®	1	1	1	1	1
Unemployed	0.7 (0.3–1.6)	0.7 (0.4–1.5)	1.0 (0.5–2.0)	1.0 (0.5–1.8)	0.8 (0.4–1.7)
**Smoking status**					
Non-smoker ®	1	1	1	1	1
Smoker	1.0 (0.4–2.4)	0.4 (0.2–0.9)*	0.8 (0.4–1.7)	0.9 (0.4–1.7)	1.9 (1.0–3.7)*

The following variables were entered into the multivariable logistic regression: gender, employment status, age, marital status, education level, and smoking status. ® Reference categories.

* Statistically significant.

### Acceptance for the inclusion of health warnings on cigarette sticks

The majority of participants (71.6%) ‘agreed’ or ‘strongly agreed’ to the inclusion of health warnings on cigarette sticks, whereas around 10% ‘disagreed’ or ‘strongly disagreed’, while 17.5% were ‘neutral’ or undecided. Non-smokers were three times more likely to support the addition of health warnings on cigarette sticks compared to current smokers (OR=3.1; 95% CI: 1.6–6.1, p=0.001), indicating a significant smoking status effect. Respondents who had secondary education and lower were almost two times more likely to support the inclusion of health warnings than those who had a university education and higher (OR=1.9; 95% CI: 1.02–3.7, p=0.042).

### Qualitative results

*Current packaging warnings*

Several comments on the packaging warnings highlighted the issue of desensitization to the warnings. Others recommended including religious messages and finding other ways to quit smoking:

*‘I do not pay attention to it, so I can smoke.’* (Male, 26 –45 years, daily smoker)

*‘Religious messages and their impact are the deepest reminders that it is forbidden and destructive for the person, his family, and society.’* (Female, 26–45 years, non-smoker)

*Theme of statistics on mortality*

There were some discrepancies between respondents’ perceptions of using this theme on cigarette sticks: some perceived that it would be better to use more powerful words, while others perceived that the messages should be focused on the benefits of quitting rather than mentioning the negative consequences of smoking:

*‘Please write more powerful words.’* (Female, 26–45 years, non-smoker)

*‘Discouraging non-smokers from smoking is possible if the warning is external and clear from the outside, but if the warning is internal, as in the picture, usually, those who are willing to smoke give one of their friends a cigarette to experiment so it will be inadequate and meaningless.’* (Male, 18–25 years, non-smoker)

*‘Messages should have more of a role in desirability than disadvantages.’* (Female, 18–25 years, non-smoker)

*Theme of supportive messages*

Some participants preferred using warning messages:

*‘I’d rather have warning words.’* (Female, 18–25 years, non-smoker) (Table 5)

**Table 5:** Summarizing the main qualitative data of each method used: A cross-sectional survey, Al-Madinah, 2023 (N=285)

*Warning methods*	*Main findings*
**Cigarette packaging warnings**	Some of the responders perceived that the smoker ignores the pictures and warnings on the cigarette packaging. Others perceived that the warnings were not effective and believed in finding other effective ways to quit smoking.
**Cigarette stick warnings**	
Statistics on mortality theme	Most of the respondents perceived that these warnings were effective. However, some responders perceived minimal effects regarding these words used in the warnings and needed stronger words to make them more effective.
Health consequences theme	The majority of the respondents perceived that these warnings about the health impacts were effective, and it is a good idea to include them.
Social and financial consequences theme	Some of the responders perceived that these messages were effective.
Supportive messages to quit theme	Although some of the responses were positive towards using these messages, others perceived it to be ineffective and that using warning messages would be more beneficial compared to the supportive messages.

*Acceptance for the inclusion of health warnings on cigarette sticks*

Most of the comments were positive for the inclusion of health warnings on cigarette sticks. Nevertheless, some perceived that smoking cannot be stopped unless the individual has the intention to quit:

*‘Smokers can only quit if they have the will because if they want to smoke, they won’t hear or see warnings.’* (Male, 46–65 years, daily smoker)

## DISCUSSION

This study aimed to evaluate perceptions of the effectiveness of current cigarette packaging warnings and health-related warnings on individual cigarette sticks. We found that the warnings used on the packaging were mostly perceived as ineffective, whereas novel warnings on individual cigarette sticks were considered more effective in preventing non-smokers from starting smoking. In addition, we found that non-smokers support the addition of health warnings on cigarette sticks more than current smokers. An online survey conducted in four countries, Canada, Australia, the United States, and the United Kingdom, indicated that warnings on cigarette packaging had a small effect in encouraging smokers to quit smoking, which is consistent with our findings. On the other hand, the warnings on cigarette sticks, which explained the financial costs of smoking and its effects on others, were rated the most effective of all themes used^23^. Another online study found that around half of Australians believed that packaging warnings were less effective, similar to our estimate of 52.3%, which might be a result of smokers’ desensitization and self-exemption^24^.

Due to banning strategies implemented by tobacco control agencies, tobacco companies are compelled to pay closer attention to the cigarette’s packaging and sticks, to better communicate their brand’s image and to circumvent laws restricting smoking. The sticks are being used for communicative purposes in different ways: Smith et al.^26^ investigated seven methods used by tobacco companies to utilize cigarette sticks as a marketing tool, including: brand name, image or logo; text descriptors such as ‘light’ or ‘silver’; colors, designs, and symbols; and filter enhancements. Furthermore, Smith et al.^26^ recommend regulations that consider the communication potential of cigarette sticks and packs, to achieve effective control of cigarette marketing and promotion^26^.

A standardized cigarette policy is an innovative concept that considers the dissuasive technique together with banning flavors and adjusting nicotine, as three pillars that together form an effective tool for preventing tobacco companies from getting around marketing restrictions^27^.

A recent scoping review on the available knowledge on dissuasion, investigated several types and approaches to dissuade the use of cigarettes. The review concluded that the dissuasive approach is a promising tobacco control strategy. The review also identified a gap in the research, wherein all the studies were conducted in North America, Europe, Australia, and New Zealand. The current study tries to fill this gap by investigating the perceptions of the effectiveness of dissuading the use of cigarettes in a Middle Eastern population^28^.

### Limitations

This study has some limitations. First, due to the use of an online survey, selection bias could not be excluded, and the generalizability of the results to all the population of Saudi Arabia may be affected. Furthermore, although a convenient sampling technique was utilized, which could affect population representativeness, the sample characteristics were approximate of the population regarding some independent variables such as gender distribution. Moreover, only the perceived intentions towards dissuasion of cigarettes were measured, not the actual behaviors of the respondents, due to the novelty of the subject. Finally, the utilization of digital photographs instead of tactile materials might have had an impact on participants’ answers.

### Implications

Current cigarette packaging warnings in combination with novel warnings on individual cigarette sticks, are considered helpful strategies for smoking control. Because of the current research gap in exploring the effectiveness of current packaging on dissuasion of cigarette use, more studies are recommended to investigate the perception of several types of dissuasive methods at the national level, which will add more valuable information to this field. Moreover, we recommend that health authorities consider the implementation of new methods of dissuasion of cigarette use.

## CONCLUSIONS

Most participants perceived that package warnings were ineffective, but warnings on cigarette sticks were effective methods of dissuasion of cigarette use. Smokers were almost twice as likely to perceive as effective supportive messages to quit than non-smokers. The majority of participants ‘agreed’ or ‘strongly agreed’ to the inclusion of health warnings on cigarette sticks.

## Data Availability

The data supporting this research are available from the authors on reasonable request.
